# 

**Published:** 1820-10

**Authors:** 


### MONTHLY CATALOGUE OF MEDICAL BOOKS.

Views of the Muscles of the Human Body; containing eighteen Plates, drawn from Nature, and engraved by Mr. G. Lewis; accompanied by suitable explanatory References. Designed as a Guide to the Student of Anatomy, and a Book of Reference for the Medical Practitioner. 4to. 1l. 11s. 6d.

Appendix to a Catalogue of Books on Anatomy, Medicine, Surgery, Midwifery, Materia Medica, Chemistry, Botany, Veterinary Surgery, &c. containing all the new Works from September 1818 to October 1820; also a few Second-hand Books; sold by Highley and Son, Medical Booksellers, 174, Fleet-street. To which is added, a List of all the Lectures delivered in London, with the Terms, Hours of Attendance, &c.; also the Pay of the Medical Department of the Army, Navy, and East-India Company's Service. 1s.

Lectures on the Structure and Physiology of the Parts composing the Skeleton, and on the Disease of the Bones and Joints of the Human Body; preceded by some Observations on the Influence of the Brain and Nerves; delivered before the Royal College of Surgeons of London, in the year 1820. 8vo. 12s.

Few Hints, relative to Cutaneous Complaints. A new Edition; by T. M. Kelson. With some further Remarks on the Structure and Function of the Skin; by C. Kelson, of the Royal College of Surgeons. 8vo.

Lectures on the Duties and Qualifications of a Physician; more particularly addressed to Students and Junior Practitioners. By the late John Gregory, M.D. F.R.S. &c. A new Edition. 12mo. 4s.

The Hunterian Oration, delivered before the Royal College of Surgeons in London, February 21st, 1820. By A. Carlisle. 4to. 5s.

Popular Observations on Regimen and Diet. By John Tweed. 12mo. 5s.

General Principles of Psycho-Physiology. By E. S. Pearson. 12mo. 2s. 6d.

A Treatise on Dyspepsia, or Indigestion. By J. Woodforde, M.D. 8vo. 2s. 6d.

An Essay on Mercury; wherein are presented Formulæ for some Preparations of this Metal: including Practical Remarks on the safest and most effectual Methods of administering them for the Cure of Liver Complaints, &c. By David Davies, M.D. 8vo. 2s. 6d.

Surgical Observations on the Constitutional Origin and Treatment of Local Diseases, and on Aneurism. By John Abernethy, F.R.S. Fifth Edition. 8vo. 8s.

[MESSRS. HIGHLEY AND SON.]

